# Impact of the rollout of the national social prescribing link worker programme on population outcomes: evidence from a repeated cross-sectional survey

**DOI:** 10.3399/BJGP.2024.0542

**Published:** 2025-11-04

**Authors:** Anna Wilding, Efundem Agboraw, Luke Munford, Matthew Sutton, Stewart W Mercer, Chris Salisbury, Morgan Beeson, John Wildman, Paul Wilson

**Affiliations:** 1 Health, Organisation, Policy and Economics, Centre for Primary Care and Health Services Research, University of Manchester, Manchester, UK; 2 Centre for Health Economics, Monash University, Melbourne, Victoria, Australia; 3 Centre for Population Health Sciences, Usher Institute, College of Medicine and Veterinary Medicine, University of Edinburgh, Edinburgh, UK; 4 Centre for Academic Primary Care, National Institute for Health and Care Research School for Primary Care Research, Population Health Sciences, Bristol Medical School, University of Bristol, Bristol, UK; 5 Newcastle University Business School, Newcastle University, Newcastle upon Tyne, UK; 6 Frederick Douglass Centre, Newcastle University, Newcastle upon Tyne, UK

**Keywords:** cross-sectional survey, general practice, link workers, population outcomes, primary care, social prescribing

## Abstract

**Background:**

Social prescribing link workers have been rolled out nationally through the Additional Roles Reimbursement Scheme. Link workers connect people to advice and support to address the non-medical and social issues affecting their health and wellbeing.

**Aim:**

To determine whether the rollout of social prescribing link workers through primary care networks improved population outcomes.

**Design and setting:**

Repeated cross-sectional survey of General Practice Patient Survey (GPPS; 2018 to 2023) data combined with administrative workforce data.

**Method:**

Logistic regression models were used to relate the number of full-time equivalent social prescribing link workers per 50 000 patients to five population outcomes.

**Results:**

In total, data from 4 132 676 responders from repeated cross-sections of the GPPS were used. An additional full-time equivalent link worker per 50 000 patients was associated with higher probabilities of responders with long-term conditions having confidence in managing long-term condition(s) (odds ratio [OR] 1.006, 95% confidence interval [CI] = 1.001 to 1.010) and having enough support from local services (OR 1.005, 95% CI = 1.001 to 1.008). For all responders, the same size increase in link workers was associated with a higher probability of having a good experience at their general practice (OR 1.015, 95% CI = 1.004 to 1.027). For responders with mental health needs, this increase in link workers was associated with a higher probability of having their mental health needs understood (OR 1.012, 95% CI = 1.003 to 1.021).

**Conclusion:**

The rollout of link workers was associated with small improvements in patient experience and slightly better outcomes for population groups specifically targeted for social prescribing. Future work is required to determine whether the scheme is financially sustainable and to ensure it does not widen existing health inequalities.

## How this fits in

The 2019 NHS Long Term Plan stimulated a rapid expansion of social prescribing link workers. This study found that this was associated with improved outcomes for individuals specifically targeted for social prescribing and overall patient experience with general practice. Employment of social prescribing link workers has had the intended outcomes specified in the NHS Long Term Plan. Future work is required to determine whether the scheme is financially sustainable for social prescribing as a whole and to ensure it does not widen existing health inequalities.

## Introduction

Social prescribing link workers connect people referred from primary care to sources of support in the community. These can include activities and services that address practical, social, and emotional needs affecting health and wellbeing. The 2019 NHS Long Term Plan stimulated a rapid expansion of provision with a commitment to recruit 1000 social prescribing link workers across the country by 2021.^1^ This was later boosted by a further commitment that every patient in England should have access to a social prescribing link worker by 2022.^2^ To support these commitments, funding for social prescribing link workers was made available through the Additional Roles Reimbursement Scheme (ARRS).^3^ Primary care networks (PCNs), groups of general practices working together to meet the needs of their population,^4^ could apply for reimbursement to employ people in the additional roles they perceived most important to meet the needs of their local population.

Social prescribing link workers aim to provide more positive health and wellbeing outcomes for people with long-term condition(s), who may require support for their mental health, are lonely or isolated, and/or have complex social needs.^5^ Patients can self-refer or be referred by a healthcare professional within general practice to a social prescribing link worker. The link worker will then work with the patient to identify and connect them to activities, groups, or services within their local community that align with their needs to support their overall health and wellbeing. From the NHS perspective,^5^ the intended impact is on the wider determinants of health and should ensure each patient:

- feels more in control and able to manage their own health and wellbeing;
- is more physically active;
- is better able to manage practical issues, such as debt, housing, and mobility; and
- is more connected to others and less isolated or lonely.

There is good understanding about how the social prescribing link worker role can work to support access and engagement with services.^6,7^ Qualitative research shows that patients who do engage generally report positive experiences when it meets their needs and expectations.^8,9^ However, research examining the effects of social prescribing has been suboptimal and subject to a high risk of bias.^10–13^ Much of this evidence is derived from small-scale studies limited by poor design and reporting, making it difficult to reliably judge how and in what circumstances social prescribing could deliver benefits. Only a few well-conducted, empirical evaluations of the impact exist^14–17^ and have so far failed to demonstrate improved health and wellbeing for service users, but suggest that finding ways to improve access and engagement with services may lead to better overall outcomes.

To date, research on the rollout of ARRS-funded social prescribing link workers has focused on examining how provision expanded,^18^ showing that areas with the highest reported need had lower levels of provision of social prescribing link workers and suggesting that an inverse care law exists in social prescribing.^19^

This study adds to this literature by estimating the associations between ARRS-funded social prescribing link workers and population outcomes. Outcomes were selected for the study that reflected the stated NHS aims for social prescribing^5^ from a nationally representative survey conducted with patients from all general practices in England. The associations were then estimated between the rollout and these outcomes, and sensitivity analyses were conducted to test the robustness of the results.

## Method

### Patient questionnaires

Data from the annual General Practice Patient Survey (GPPS) were used in the study. This was sent to a sample of individuals registered with each general practice in England between January and March and received around 700 000 responses each year.^20^ Data from 6 years of the survey were used (2018–2023) covering the periods before and after the introduction of the social prescribing link worker expansion. Supplementary Table S1 contains the response rate for each survey year. Alongside questions on service use and experience, it asked responders about their age, gender, ethnicity, and employment status. Area-level measures of deprivation (measured by the income domain of the Indices of Deprivation^21^) and rurality (measured by the Office for National Statistics rural–urban classifications^22^) are also available.

### Population outcomes

The following outcomes were selected from the GPPS:

- confidence in managing long-term condition(s);
- support from local services and organisations;
- not feeling isolated from others;
- having a good experience at their general practice; and
- having their mental health needs understood by a healthcare professional.

These were either closely related to the stated aims set out by the NHS^5^ or reflected two further hypothesised effects of social prescribing. The first of these was that social prescribing link workers would improve patient experience by contributing to better access and availability, and alleviation of GP pressure.^23–25^ The second possible effect was on helping to address mental health needs,^12^ because this was one of the target health conditions for this initiative.^5^ The outcomes are summarised in Box 1, along with the aim or hypothesised effect to which they are linked. Supplementary Table S2 contains the hypothesised impact of social prescribing link workers on outcomes, whether these effects could be direct or indirect, and the reasoning underpinning these expected effects.

**Box 1.** Outcomes assessed within the General Practice Patient Survey^a^

Variable	Question	Coded as positive (= 1)	Coded as negative (= 0)	Exclusions
**NHS aim: feels more in control and able to manage their own health and wellbeing**				
**Confidence managing LTC**	How confident are you that you can manage any issues arising from your condition (or conditions)?	Very confident or Fairly confident	Not very confident or Not at all confident	No LTC or Not applicable or Don't know
**NHS aim: better able to manage practical issues, such as debt, housing, and mobility**				
**Support**	In the last 12 months, have you had enough support from local services or organisations to help you to manage your condition (or conditions)?	Yes, definitely or Yes, to some extent	No or I haven't needed support	No LTC or Not applicable or Don't know
**NHS aim: is more connected to others and less isolated or less lonely**				
**Not isolated**	Have you experienced any of the following over the last 12 months?: Problems with your physical mobility Two or more falls that have needed medical attention Feeling isolated from others None of these	Any option(s) other than 'Feeling isolated from others'	Feeling isolated from others	No options selected
**Alleviate general practice pressures**				
**Good GP experience**	Overall, how would you describe your experience of your GP practice?	Very good or Fairly good	Neither good nor poor, Fairly poor, or Very poor	—
**Address mental health needs**				
**Mental health needs understood**	During your last general practice appointment, did you feel that the healthcare professional recognised and/or understood any mental health needs that you might have had?	Yes, definitely or Yes, to some extent	No, not at all	Did not apply to my last appointment or I did not have any mental health needs

^a^All unanswered or multi-coded responses are excluded. LTC = long-term condition.

### Social prescribing link worker employment

Administrative data on social prescribing link worker employment from the primary care workforce quarterly update,^26^ which collates data from PCN ARRS claims. Because most social prescribing link workers are employed to work across practices within a PCN, the availability of social prescribing link workers at the PCN level was measured in this study. To account for variation in PCN size, full-time equivalent (FTE) social prescribing link workers per 50 000 patients registered with practices in the PCN were calculated. This was the average population size of a PCN. Given that the GPPS was completed from January to March, the survey responses were linked to staff numbers as at the end of the previous December in the current study.

### Statistical methods

The rollout of ARRS-funded social prescribing link workers can be treated as a natural experiment. This type of methodology has been used in previous public policy evaluations where national rollout was not under the control of the research team.^27,28^ All PCNs start to be ‘exposed’ at the same time, but the level of social prescribing link worker employment varies both over time and place. The measure of exposure, therefore, changes with an increasing trend in the number of social prescribing link workers over time and variations across PCNs in the numbers of social prescribing link workers they employ. This aggregate measure of exposure was related to population outcomes.

As the outcomes are binary, logistic regression models were estimated and included year indicators plus information on responders’ age, gender, ethnicity, employment status, deprivation, and rurality (the full list of variables is shown in Supplementary Table S3). The magnitude of the estimated effects are presented in two ways, first as odds ratios (ORs) and second as average marginal effects showing the changes in outcomes associated with one additional FTE social prescribing link worker per 50 000 patients.

The dataset is structured as a series of cross-sections of responders nested within general practices that are themselves nested within PCNs. The standard errors are clustered at the PCN level, as this is the level at which exposure is determined.^29^ All analyses were conducted in Stata (version 18) using the *logistic* command. Further details of the statistical analysis are shown in Supplementary Box S1.

The NHS Direct Enhanced Services contract^5^ specifies some specific target populations for social prescribing. Three sets of models were run, some including all responders and some focusing only on responders in these target groups. These are summarised in Box 2, with further details provided in Supplementary Tables S4 and S5.

**Box 2.** Samples used for each model and outcome

Model	Outcome				
	Confidence managing LTC	Support	Not isolated	Good GP experience	Mental health needs understood
**1**	n/a	n/a	All responders	All responders	n/a
**2**	Responders with LTC(s)	Responders with LTC(s)	Responders with LTC(s)	Responders with LTC(s)	Responders with LTC(s) and mental health needs
**3**	Responders with mental health needs and LTC(s)	Responders with mental health needs and LTC(s)	Responders with mental health needs	Responders with mental health needs	Responders with mental health needs

LTC = long-term condition. n/a = not applicable because no model estimated.

### Supplementary analyses

In the first supplementary analysis, the relationship between population outcomes and other direct patient care roles introduced in the ARRS alongside social prescribing link workers were considered. These other roles included: pharmacists (including those in training), health and wellbeing coaches, care coordinators, mental health practitioners, first-contact physiotherapists, paramedics, nursing associates (including in training), occupational therapists, physician associates, and advanced practitioners. PCNs may opt to employ these staff over social prescribing link workers or alongside them to meet both clinical and wider needs of their patients, and some of the outcomes measured could be because of employment of these roles instead of, or in addition to, link workers.

As the ARRS represents a large increase in roles within primary care and social prescribing link workers represent a small but significant proportion of that increase, a supplementary analysis was run that assessed the associations between outcomes and the scheme as a whole (including the total FTE for all roles hired under the ARRS) and the proportion of those roles that are social prescribing link workers.

The main analysis assumed the constant effects of social prescribing link worker provision. In the next supplementary analysis, the study allowed for the ORs of social prescribing link workers to vary by deprivation level, measured by deciles of income deprivation. This was undertaken in two ways: initially, through an interaction model, and then by subgroup analysis when the authors identified a significant variation by deprivation.

## Results

### Summary statistics

The analysis sample included 4 132 676 responders from 6991 general practices in 1268 PCNs. Descriptive statistics for the population, the GPPS questionnaire sample, and the samples used to analyse each outcome in Supplementary Table S4 were compared. The survey is broadly representative of the population, but with a higher proportion of responders from older age categories, from White ethnic groups, whose gender was female, and those living in rural areas.

**Figure 1.**
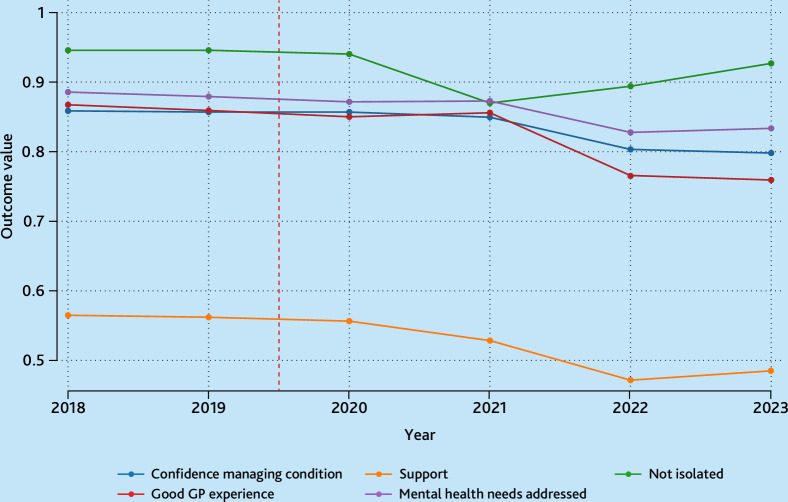
Mean values of outcomes, 2018–2023. Vertical red dotted line indicates when the Additional Roles Reimbursement Scheme was introduced.

Figure 1 shows the annual trends in average outcomes across responders in each year. The red dotted line indicates when the ARRS was introduced. The average outcomes were stable before the ARRS and decreased during the COVID-19 pandemic. By 2023, average outcomes had stabilised at a lower level, except for the percentage of responders not feeling isolated, which returned to the pre-COVID-19 level. The raw outcome responses are summarised in Supplementary Table S5.

Supplementary Figure S1 shows annual trends in social prescribing link workers across the study period based on workforce data from 1268 PCNs. The box plots indicate an increase over time and high variability in FTE social prescribing link workers per 50 000 patients between PCNs in each year.

### Main findings

Table 1 presents the associations of social prescribing link worker provision with the five population outcomes. All ORs in the main models were positive and significantly different from one (Model 2: confidence managing long-term conditions and support, Model 1: good GP experience, and Model 3: mental health needs understood, with *P*<0.05), with the exception of not feeling isolated from others (Model 1). They ranged from 1.005 (95% confidence interval [CI] = 1.001 to 1.008) for having enough support from local services (Model 2) to 1.015 (95% CI = 1.004 to 1.027) for a good experience at their general practice (Model 1).

**Table 1.** The association of one FTE link worker per 50 000 patients on population outcomes^a^

Model	Confidence managing LTC	Support	Not isolated	Good GP experience	Mental health needs understood
**1: all responders**					
Odds ratio (95% CI)	—	—	1.001 (0.996 to 1.005)	1.015 (1.004 to 1.027)	—
*P*-value	—	—	0.74	0.009	—
Average marginal effect, pp	—	—	0.01	0.21	—
Mean outcome value, %	—	—	92.0	82.8	—
Observations, *N*	—	—	4 132 676	4 176 144	—
**2: responders with at least one LTC**					
Odds ratio (95% CI)	1.006 (1.001 to 1.010)	1.005 (1.001 to 1.008)	1.001 (0.996 to 1.006)	1.015 (1.003 to 1.027)	1.013 (1.003 to 1.022)
*P*-value	0.01	0.02	0.65	0.01	0.009
Average marginal effect, pp	0.07	0.11	0.01	0.20	0.15
Mean outcome value, %	83.8	52.9	89.4	83.3	86.0
Observations	2 299 456	2 280 909	2 422 598	2 451 230	1 037 073
**3: responders who declared a mental health condition**					
Odds ratio (95% CI)	1.004 (0.998 to 1.010)	1.004 (0.999 to 1.009)	0.999 (0.994 to 1.004)	1.013 (1.001 to 1.025)	1.012 (1.003 to 1.021)
*P*-value	0.16	0.14	0.75	0.04	0.006
Average marginal effect, pp	0.06	0.09	−0.01	0.17	0.14
Mean outcome value, %	77.9	59.8	86.8	83.3	86.1
Observations	970 385	961 748	1 618 935	1 642 426	1 651 360

^a^The 95% confidence intervals are based on clustered standard errors at PCN (*n* = 1268) level. Models include individual- and area-level adjustment factors; these are listed in Supplementary Table S3. Average marginal effect (AME) represents the increase in the probability of achieving the outcome for a one unit increase in FTE link worker per 50 000 patients. To estimate the percentage point, the AME is multiplied by 100. FTE = full-time equivalent. LTC = long-term condition. PCN = primary care network. pp = percentage point.

For an average responder with a long-term condition(s), these associations represent improvements in outcomes of 0.07 percentage points (pp) for probability of reporting confidence in managing long-term condition(s) and 0.11 pp in the probability of reporting having enough support from local services. For an average responder, there was a 0.21 pp increase in the probability of reporting a good experience at their general practice. For an average responder with mental health needs, the probability of reporting their needs were understood increased by 0.14 pp (Table 1).

The findings remain robust and qualitatively the same when the models are limited to patients with long-term conditions only (Table 1, Model 2). This suggests that, for outcomes such as good GP experience and mental health needs addressed, the average marginal effects are the same for those with or without long-term conditions. Similarly, when the analysis is limited to patients who report mental health needs (Table 1, Model 3), the results remain qualitatively the same. For two outcomes, confidence in managing long-term condition(s) and support from local services, the results become not statistically significant when limited to those with long-term conditions. This may be because of the reduced sample size.

Unadjusted models are provided in Supplementary Table S6 and demonstrate that the ORs and *P*-values are similar without covariate adjustment. Supplementary Table S3 shows the effects of the covariates included in the models in Table 1.

Table 2 quantifies these associations for an average-sized PCN and for the national population. Employing an additional FTE social prescribing link worker in an average PCN would be associated with 56 more people feeling supported, 71 more people reporting that their mental health needs were understood, and 104 more people reporting a good experience of their GP practice. Scaled up to the national population, these results represent improvements for approximately 70 000, 90 000, and 130 000 people, respectively.

**Table 2.** Estimated number of additional patients with positive outcomes associated with an additional social prescribing link worker per 50 000 population in an average and in all PCNs^a^

Model, *n*	Confidence managing LTC	Support	Not isolated	Good GP experience	Mental health needs understood
**1: all responders**					
In an average PCN	—	—	0	104	—
In all PCNs	—	—	0	131 872	—
**2: responders with at least one LTC**					
In an average PCN	37	56	5	98	75
In all PCNs	46 916	71 008	6340	124 264	95 100
**3: responders who declared a mental health condition**					
In an average PCN	32	47	−4	83	71
In all PCNs	40 576	59 596	−5072	105 244	90 028

^a^The PCN effect is calculated by multiplying the average marginal effect by the exposure scale, in this case 50 000 population, which reflects the average PCN size (see Supplementary Table S7). The population effect is then calculated by multiplying the PCN effect by the number of PCNs in England in 2023 (*n* = 1268). LTC = long-term condition. PCN = primary care network.

### Supplementary analysis

#### Inclusion of other direct patient care roles employed under the ARRS

Statistics on the rollout of these roles are given in Supplementary Figure S2 and Supplementary Table S7. The findings for social prescribing link workers remain robust at the same significance and magnitude (see Supplementary Table S8).

#### ARRS as a whole and proportion of social prescribing link workers

The results (see Supplementary Table S9) highlight a small positive association with the number of FTE ARRS roles for three out of five outcomes (support, good experience, and mental health). However, it is inconsistent across models. For PCNs with a higher proportion of social prescribing link workers, there is a significant positive association with confidence in managing long-term condition(s) (Model 2) and not being isolated (Model 1), with the remaining outcomes being insignificant. This model supports the notion that the study was assessing the impact of social prescribing in its main model (Table 1) rather than an increased number of roles within primary care.

#### Association with health inequalities

No significant differences in the ORs of social prescribing link workers across the level of area deprivation were found for outcomes good GP experience (Model 1) and mental health needs understood (Model 3) (see Supplementary Table S10). For having enough support from local services (Model 2), the study found some evidence that the least deprived areas had significantly higher ORs than the most deprived areas. These ORs in the subgroup analysis in Supplementary Table S11 are positive and do not provide evidence that they vary across deciles. For confidence in managing long-term condition(s) (Model 2) and not being isolated (Model 1), the more deprived areas had larger positive ORs. Within the subgroup analysis, the ORs demonstrate effects in favour of less deprived areas for not being isolated and for confidence in managing long-term condition(s).

## Discussion

### Summary

The number of social prescribing link workers employed through the ARRS was associated with small positive changes in population outcomes that were aligned with the NHS’s specified aims for social prescribing.^5^ There was also an improvement in the overall experience that all responders reported with their general practice.

The ORs were small in magnitude at the individual level but represent meaningful associations at the population level. For context, by March 2023, 3.2% of the registered GP adult population had been referred to social prescribing,^30,31^ so any effect on responses to the national GPPS will be very diluted. The findings suggest that an additional FTE social prescribing link worker per 50 000 patients in all PCNs (approximately one extra link worker per average PCN) was associated with an increase nationally in approximately 47 000 people reporting confidence in managing long-term condition(s) and 132 000 people reporting having a good GP experience.

### Strengths and limitations

This is the first study, to the authors' knowledge, to quantitatively estimate the effects of the national rollout of social prescribing link workers in England, with a large sample size of >4 million individuals from 2018 to 2023. This scale makes it possible to estimate associations on population outcomes robustly. Previous systematic reviews have highlighted that the current evidence is suboptimal when it comes to estimating the impact of social prescribing.^10–13^ This study addresses concerns of small sample sizes, lack of counterfactuals,^10^ and uncontrolled studies with short timeframes.^11^ The outcomes — confidence in managing long-term condition(s), support, and not feeling isolated — align with three of the four NHS aims for social prescribing^5^ and additional related themes — alleviating pressure in GP services and mental health needs. This allowed the estimation of the direct and indirect associations of social prescribing link worker employment in a PCN.

The use of administrative records of workforce data was an additional strength. This contained FTE social prescribing link workers (and other roles) hired under the ARRS, which were scaled to account for PCN population size. This meant it was possible to determine the associations of the varying levels of provision across PCNs. The study evaluated the national rollout as a natural experiment to assess the relationship between an exogenous increase in social prescribing link workers and population outcomes. The results were further strengthened by including other direct patient care roles under the ARRS within the models.

Despite some key strengths, there were limitations to the study findings. First, repeated cross-sectional data were used instead of longitudinal data. This meant the study was not following the same responders across the study period. Second, it was not known whether an individual responder to the survey had been referred to a social prescribing link worker. Finally, there could be a non-linear relationship of the provision of social prescribing link workers, such that outcomes do not continue to improve as much at higher levels of link worker provision. The regression models were re-estimated allowing for this non-linearity and the results were very similar.

### Comparison with existing literature

The current study addresses previous calls for the need for large-scale, robust evidence to support social prescribing link workers.^10,13^ The positive ORs found in the current study from the rollout of social prescribing link workers adds to the evidence from the few robust empirical evaluations conducted previously.^14–17^ This was particularly true in managing mental health conditions,^14^ as the current study found that higher numbers of social prescribing link workers was associated with higher probabilities of having mental health needs addressed. The study did not use self-reported measures of physical or mental health,^15^ objective health measures,^17^ or healthcare cost-savings,^16^ as in previous literature. However, the findings of patients having increased confidence in managing conditions and improvement in overall GP experience suggest this may be associated with relative improvements in physical or mental health and quality of healthcare services. This is on the hypothesised pathway of social prescribing link workers — patients have better management of their condition,^17^ which reduces healthcare utilisation.^16^

### Implications for research and practice

The results indicate positive associations with social prescribing at a population level. Areas with higher provision had improved outcomes, which was robust when other roles funded via the ARRS and thought to have an impact on patients’ outcomes were included. This provides evidence to support the usefulness of social prescribing link workers; however, some key factors need to be addressed regarding sustainability. The ARRS only reimburses the link worker role, which involves directing patients to services and activities in the community that aim to improve their health and wellbeing. These services are generally provided and funded by the voluntary and charity sector and, in part, by local authorities. These sites will require extra funding to meet the increased demand that social prescribing poses. This is another factor for determining long-term sustainability and whether the scheme is cost-effective. It was estimated in 2022/2023 the scheme cost the NHS £130 million;^26,32^ however, this underestimates the total cost as it does not include the cost of the onward referral destinations. For future evaluations, understanding the total cost of social prescribing would provide pivotal evidence for long-term sustainability.

The findings were unable to support the NHS aim for patients to feel more connected, less lonely, or less isolated. This could be owing to the wording of the question within the GPPS. Further qualitative or quantitative work should explore this, as isolation is a known predictor of mortality.^33^

The study was unable to identify who was engaging with social prescribing. Past evidence has highlighted that those from higher socioeconomic status engage in community activities similar to social prescribing.^34^ To ensure that social prescribing does not contribute to known health inequalities, future work should assess whether access has been equitable by evaluating patient characteristics of referrals.

Finally, policymakers and commissioners are interested in whether social prescribing referrals reduce unplanned hospital admissions. This study was unable to shed light on this outcome. Future findings should use linked healthcare records to determine whether and for whom the scheme has had the intended effects.

In conclusion, the rollout of social prescribing link workers was associated with small improvements in outcomes for individuals specifically targeted for social prescribing and had spillover on improving the overall experience at general practices. This supports the NHS’s intended patient outcomes for social prescribing link worker provision as set out in the NHS Long Term Plan. Future work is required to determine the economic sustainability of social prescribing as a whole and to ensure it does not widen existing health inequalities.

## Data

Owing to data-sharing restrictions, the data used in this study cannot be shared directly. Researchers may, however, apply to use the General Practice Patient Survey linked with other publicly available health datasets, such as primary care network workforce data, Office for National Statistics rural–urban classifications, and the Index of Multiple Deprivation.
